# Identification of long non-coding RNA using single nucleotide epimutation analysis: a novel gene discovery approach

**DOI:** 10.1186/s12935-022-02752-2

**Published:** 2022-11-04

**Authors:** Mohammad Amin Kerachian, Marjan Azghandi

**Affiliations:** 1 Medical Genetics Research Center, Mashhad University of Medical Sciences, Mashhad, Iran; 2 Department of Medical Genetics, Faculty of Medicine, Mashhad University of Medical Sciences, Mashhad, Iran; 3 Cancer Genetics Research Unit, Reza Radiotherapy and Oncology Center, Mashhad, Iran; 4 Department of Chemistry and Biology, Toronto Metropolitan University, Toronto, ON Canada; 5 Department of Animal Science, Faculty of Agriculture, Ferdowsi University of Mashhad, Mashhad, Iran

**Keywords:** Long non-coding RNA, Epigenetic, LINC02892, Exosome, Intergenic, Discovery

## Abstract

**Background:**

Long non-coding RNAs (lncRNAs) are involved in a variety of mechanisms related to tumorigenesis by functioning as oncogenes or tumor-suppressors or even harboring oncogenic and tumor-suppressing effects; representing a new class of cancer biomarkers and therapeutic targets. It is predicted that more than 35,000 ncRNA especially lncRNA are positioned at the intergenic regions of the human genome. Emerging research indicates that one of the key pathways controlling lncRNA expression and tissue specificity is epigenetic regulation.

**Methods:**

In the current article, a novel approach for lncRNA discovery based on the intergenic position of most lncRNAs and a single CpG site methylation level representing epigenetic characteristics has been suggested.

**Results:**

Using this method, a novel antisense lncRNA named LINC02892 presenting three transcripts without the capacity of coding a protein was found exhibiting nuclear, cytoplasmic, and exosome distributions.

**Conclusion:**

The current discovery strategy could be applied to identify novel non-coding RNAs influenced by methylation aberrations.

**Supplementary Information:**

The online version contains supplementary material available at 10.1186/s12935-022-02752-2.

## Introduction

Long non-coding RNAs (lncRNAs) comprise different species of RNA which exceed 200 nucleotides that are not usually translated into proteins (limited protein-coding capacity) [1]. They modulate the gene expression at various levels, including transcriptional, post-transcriptional, and epigenetic processing [2, 3]. Additionally, growing evidence has revealed that lncRNAs could play an important role in various cancers by regulating oncogenes or tumor-suppressors, or even harboring oncogenic and tumor-suppressing effects, representing a new class of cancer biomarkers and therapeutic targets [4–8]. Dysregulation of lncRNAs normally affects cellular functions such as apoptosis resistance, cell proliferation, tumor suppressor evasion, metastasis promotion, and angiogenesis activation in tumorigenesis [9–11], reported in breast cancer [12], glioblastoma [13], liver cancer [14], leukemia [15], colorectal cancer (CRC) [6] and several other cancers [16]. Their expression and function can be influenced by mutation [17] or epigenetic changes, including DNA methylation [8]. Epigenetic modifications have key roles in cancer biology and cell growth [18–20]. Recent studies of DNA methylation analysis in tumor cells have identified several thousand differential methylated regions (DMRs) [21] with less than 3% mapped to promoters. The majority of DMRs are found in introns or intergenic regions [22]. It is widely known that tumor cells display global demethylation of intergenic regions expressing large hypomethylation across different types of tumors [21, 23–25]. Of note, one potential function of intergenic DMRs is to regulate the non-coding RNA (ncRNA) expression [22]. It is predicted that more than 35,000 ncRNA especially lncRNA are positioned at the intergenic regions [26]. Emerging research indicates that one of the key pathways controlling lncRNA expression and tissue specificity is epigenetic regulation [27, 28]. Similar to germline genetic mutations, constitutive aberrant methylation may serve as the first hit (according to Knudson’s model of tumor development) in patients with cancer [29] especially at the intergenic regions. Changes in methylation could be due to single CpG methylation errors at different positions [30].

We have previously suggested an algorithm to identify methylated CpG sites (accessible in GitHub through the following link: https://github.com/Genetics-Research-Laboratory-RROC/Candidate_Primer_Region_Finder) using methylation-sensitive high resolution melting (MS-HRM), on data from methylation next-generation sequencing (mNGS). It is feasible that methylation aberrations in crucial single CpG sites could impact the function of the lncRNA similar to single nucleotide polymorphisms (SNPs) of lncRNAs, leading to different impacts on its expression and function [31–33]. Therefore, in this article based on the intergenic position of lncRNAs and single CpG site methylation, an approach for novel lncRNA discovery linked to tumorigenesis is suggested. The newly discovered lncRNA would be attributed to the analyzed cancer type. Furthermore, we used bioinformatics tools and laboratory experiments to identify and validate the novel lncRNAs.

## Materials and methods

### Identification and validation of single CpG epimutation

Single CpG epimutations were identified by mNGS [34] and verified by MS-HRM assay. Briefly, a CpG site discovery step was performed based on unbiased methylome sequencing using SureSelectXT Methyl-Seq in CRC and control groups (six individuals each) using an algorithm to identify methylated CpG sites accessible in GitHub through the following link: https://github.com/Genetics-Research-Laboratory-RROC/Candidate_Primer_Region_Finder. Then, specific primers for bisulfite-converted sequences were designed (MethPrime 2.0 software package) and synthesized (Metabion, Germany). Prior to use, MS-HRM assays were evaluated on methylated and unmethylated bisulfite converted control DNA and the optimal annealing temperatures were determined empirically.

For biological validation of the identified CpG sites, genomic DNA were isolated from formalin-fixed paraffin-embedded )FFPE( (40 cancerous and 40 normal colon tissues) and fresh (28 cancerous and 28 normal colon tissues) samples using QIAamp DNA FFPE Tissue Kit and QIAamp Fast DNA Tissue kit, respectively (Qiagen, Germany). All patients gave written informed permission to retain and analyze their samples for purposes of this study. The procedures and protocols in the present study were approved by the regional ethics committee. Subsequently, DNA was bisulfite-converted using EpiTect Fast Bisulfite Conversion Kit (Qiagen, Germany) according to the manufacturer’s instructions and amplified using the LightCycler 96 (Roche, Mannheim, Germany).

## Identification of novel long non-coding RNA

### RNA-Seq data analysis

RNA-Seq dataset for normal and colon cancer was obtained from the NCBI Sequence Read Archive (SRA) database (http://www.ncbi.nlm.nih.gov/sra), using the accession number SRR2089755 [35]. The raw reads were processed by removing the low-quality sequences (< 10% ‘N’ bases and > 85% QA > 20 bases) and ribosomal sequences with Tophat [36]. All subsequent analyses were performed using clean reads. Clean reads were aligned to the GRCh38 reference genome using Tophat [36], during which only 2 mismatches and 2 gaps were allowed for each reading. The mapped reads were then assembled using Cufflinks [37] to identify the known and novel transcripts.

### In-silico discovery of novel lncRNA

We screened for potential lncRNAs on genome confined to the discovery CpG sites, based on the following filter criteria: (1) length > 200 nucleotides (nt); (2) open reading frame (ORF) length < 400 nt; (3) no match to PFAM protein families database [38] (E value > 1e-5); (4) iSeeRNA [39] non-coding scoreL > 0.5; and (5) the Coding Potential Assessment Tool (CPAT) [40] coding probability > 0.375; (6) removal of the transcripts mapped within the 1 kb flanking regions of an annotated gene. Gene expression level was measured by the number of uniquely mapped reads per kilobase of exon region in a gene per million mappable reads (RPKM) [41].

For annotation of the novel lncRNA, the ncRNA sequence database (RNAcentral) [42] was used to align the lncRNA to screen for any sequence homology.

### In-silico evaluating the coding potentiality of lncRNA

Among the tools for evaluating coding potential, CPAT [40], CPC (Cording-Potential Calculator) [43], and RNAcode [44] were used for the evaluation of the coding potentiality of the novel lncRNAs.

### In-silico subcellular localization

Subcellular localization of lncRNAs was predicted using iLoc-LncRNA [45] and lncLocator [46].

## Experimentally validation of the novel lncRNA

### Tissue expression of novel lncRNA

For experimental validation of the RNA-Seq results, a total RNA from 40 to 40 FFPE cases (cancerous) and control (normal) tissues, CRC cell lines (Caco-2, HCT 116, HT-29, SW480, and SW48) purchased from Pasteur Institute of Iran, were isolated using RNeasy FFPE kit (Qiagen, Germany) and AcuZol (Bioneer, South Korea), respectively. cDNA was synthesized using the RocketScript RT premix (Bioneer, Korea). The gene-specific primer targeting the novel lncRNA and *GAPDH* (as a reference gene) were designed (by primer premier 6.0 software) and synthesized (Eurofins, Germany). Reverse Transcription Quantitative PCR (RT-qPCR) reaction was carried out using HOT FIREPol qPCR mix with EvaGreen (Solis BioDyne- Estonia) on the LightCycler 96 (Roche, Mannheim, Germany) and all experiments were conducted in duplicate for each sample and performed according to the digital MIQE guidelines [47].

### Sequencing of the novel lncRNA

The full-length lncRNA was obtained using the 5’- and 3’-RACE System for Rapid Amplification of cDNA Ends (RACE) standard method [48]. PCR products were separated on a 3% agarose gel. Gel products were extracted with a Gel Extraction kit (Bioneer, South Korea), cloned into pTZ57R/T vector, and sequenced by directionally using M13 forward and reverse primers.

### Protein coding potentiality

The novel lncRNA named “Long intergenic non-protein coding RNA 2892 (LINC02892)” cDNA was synthesized from HT29 cells by RT-PCR. For the test of the protein-coding potentiality of LINC02892, the enhanced green fluorescent protein (EGFP) coding sequence was inserted into the 3’ end of the putative LINC02892 open reading frame (ORF), and the fusion gene LINC02892-EGFP was cloned into the restriction sites; Nhe I and Xho I of plasmid pcDNA3.1 (Invitrogen, California, USA). Then, plasmid transfections were performed using Lipofectamine 2000 (Invitrogen, California, USA) and GFP expression was measured by fluorescence microscopy images.

### Cellular fractionation and organelle isolation

A total of 1 × 10^6^ cells were washed twice in cold phosphate buffered saline (PBS) and then incubated in hypotonic buffer (50 mM 4-(2-hydroxyethyl)-1-piperazineethanesulfonic acid (HEPES), pH 7.5, 10 mM KCl, 350 mM sucrose, 1 mM ethylenediaminetetraacetic acid (EDTA), 1 mM dithiothreitol (DTT), and 0.1% Triton X-100) on ice for 10 min. After 5 min of centrifugation at 2,000 g, the supernatant was collected as the cytoplasmic fraction, and after additional washing, the remainder was considered as nuclear pellets, which was resuspended in lysis buffer (10 mM HEPES, pH 7.0, 100 mM KCl, 5 mM MgCl2, 0.5% NP-40, 10 µM DTT and 1mM phenylmethanesulfonyl fluoride (PMSF)) to prepare the nuclear lysate. Cytoplasmic fraction was then centrifuged in an ultracentrifuge at 100,000 *g* at 4 °C for 40 min to pellet the exosomes. The supernatant was carefully removed, and the crude exosome-containing pellets were resuspended in 1 mL of ice-cold PBS. The second round of ultracentrifugation (100,000 *g* at 4 °C for 40 min) was carried out, and the resulting exosome pellet was resuspended in 500 µL of PBS. In addition, transmission electron microscope (TEM) study was performed according to standard techniques [49] to corroborate the presence of exosomes.

## Results

The current study was inspired and extended by our previous work, in which SureSelectXT assay and methylation array observations revealed two-track methylation shifts for ‘potentially functioning’ sites like CpG islands (CGIs), CPG shores, promoters, 5’- from other ‘relatively non-functioning intergenic sites [34]. As results, the algorithm found 194 regions and the two best locations with the highest differential methyaltion rates between case and control groups were subjected for lncRNA discovery.

**Fig. 1:**
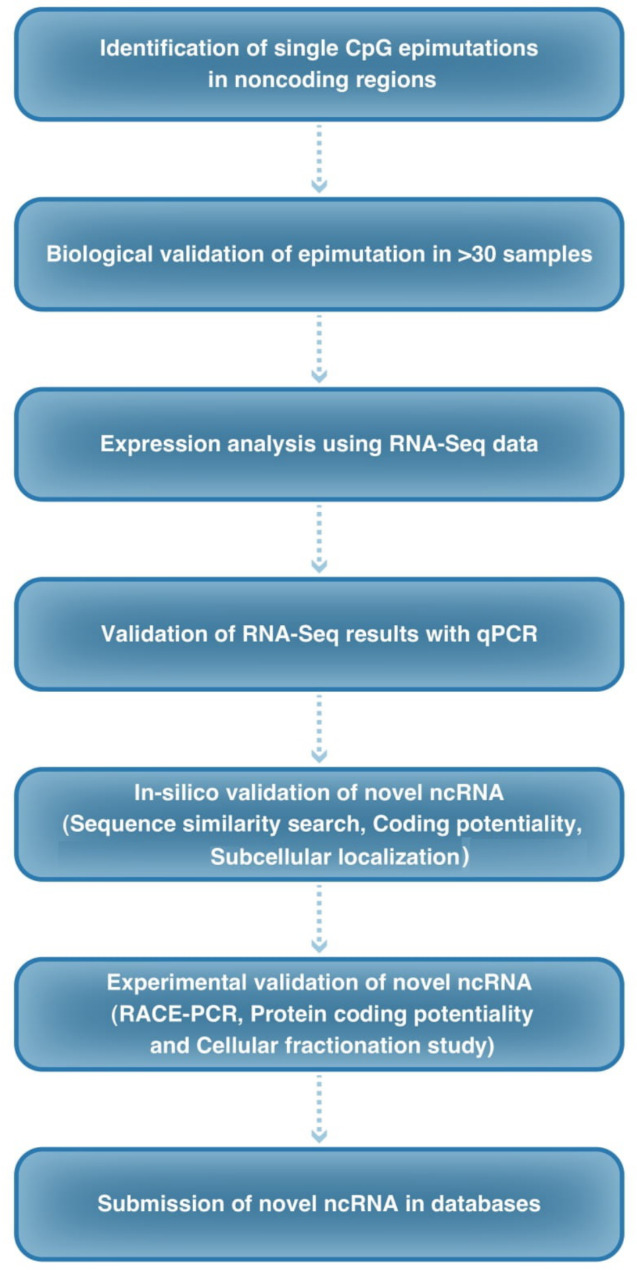
Roadmap for discovering novel lncRNA based on single epimutation

In this study, we discovered a novel lncRNA termed “LINC02892”. In order to characterize and verify the newly discovered lncRNAs, we used bioinformatics instruments and laboratory experiments to offer a path to discover lncRNA based on a single epimutation. Our path would be different with the general RNA-Seq searching publishes every day for lncRNA discovery (Fig. 1, Roadmap to detect lncRNA).

### Validation of single CpG epimutation

In our previous study, single CpG epimutations were identified by mNGS assay [50]. In order to biologically validate the mNGS results, primer sets were used to target the different regions on the bisulfite-modified DNA. Methylation-sensitive high-resolution melting assay results were in accordance with the mNGS. The real-time PCR was conducted with the LightCycler® 96 and their results were demonstrated in Supplementary Fig. 1.

### RNA-Seq data analysis and annotation of novel lncRNA

Based on a single CpG epimutation position, high-throughput RNA sequence analysis was used to identify the novel lncRNAs on genome in colon tissues (cancerous and normal). The RNA-Seq dataset for normal and colon cancer was obtained from the NCBI Sequence Read Archive database. The RNA-Seq reads were successfully mapped onto one of the CpG epimutation positions and there was no expression statement for the second CpG site.

**Fig. 2:**
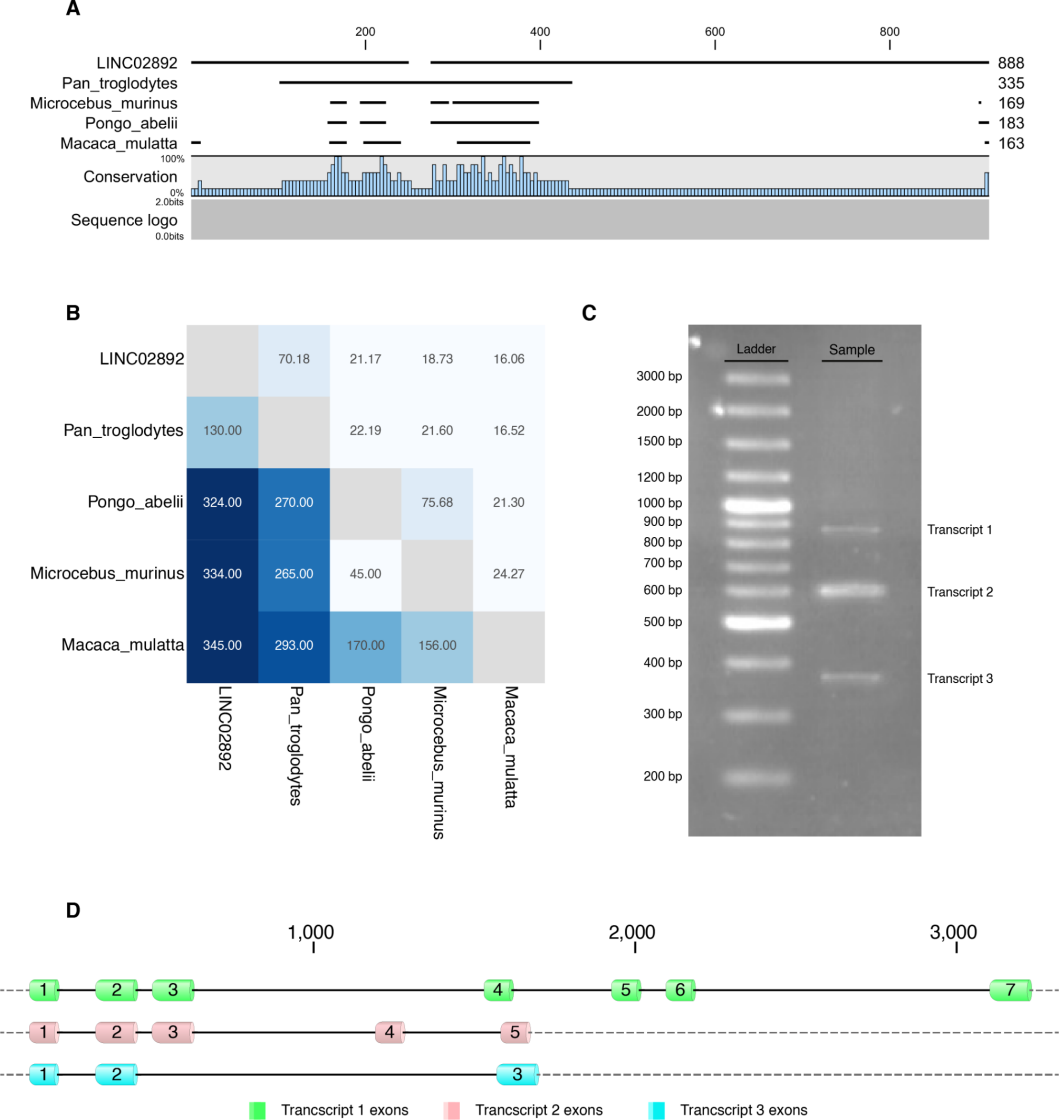
(A) Alignment of the LINC02892 sequence from humans and other organisms. (B) Pairwise comparison among complete sequences of LINC02892. The upper comparison gradient indicated the percentage identity between two sequences, and the lower comparison gradient indicated the distance between two sequences. (C) The length of LINC02892 transcripts determined by RACE PCR assays. (D) Schematic intron-exon diagram of the LINC02892 transcripts. The exons and introns are marked as boxes and lines, respectively

Our analysis with short-read mapping along with approximately 250 reads were successfully mapped onto a single CpG epimutation position on chromosome 21. The novel lncRNA, identified on chromosome 21 was further classified by comparison with the known gene annotations using RNAcentral sequence search tool. The similarity searches against a comprehensive set of ncRNAs showed that the LINC02892 sequence is similar to a long ncRNA in Pan troglodytes (Orangutan) with identity and query coverage of 70% and 79.9%, respectively (Fig. 2 A and 2B).

### 5’- and 3’-rapid amplification of cDNA ends (RACE) assay

Based on the sequence of LINC02892, the experiments of 5’- and 3’-RACE assay were initiated with total RNA from HT29 cells and resulted in three 888, 603, and 382-nucleotide (nt) antisense transcripts (Fig. 2 C), which the transcript #1 is the same as the transcript annotated with RNA-Seq data. In the current study, the three novel transcripts were identified with seven, five, and three exons, respectively (Fig. 2D). LINC02892 transcripts were submitted to NCBI under the accession numbers: Banklt2400105, LINC02892, MW248922; Banklt2400122, LINC02892, MW248923; Banklt2400131, LINC02892, MW248924; Banklt2400132, LINC02892, MW248925.

### Subcellular localization

**Fig. 3:**
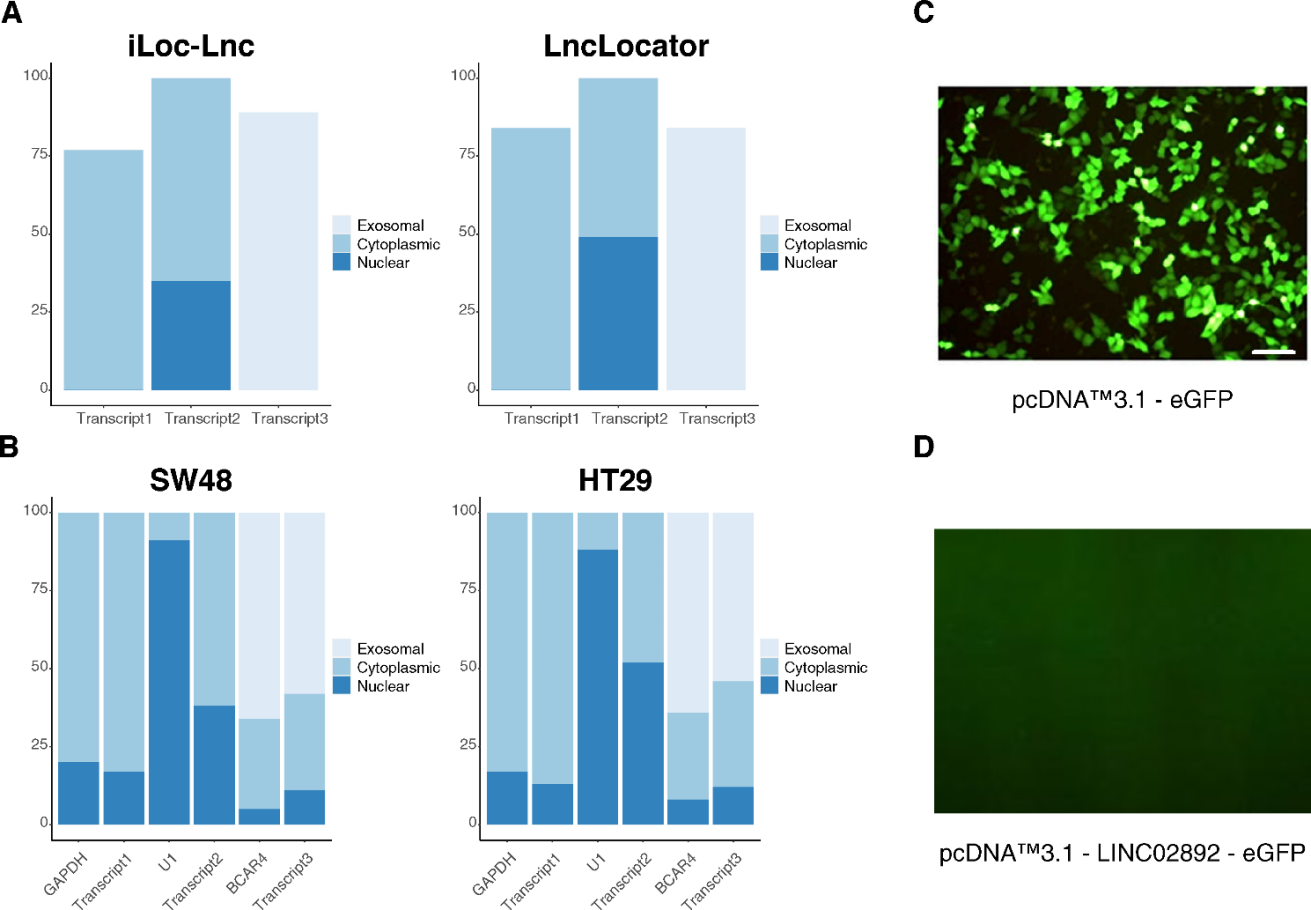
(A) In silico subcellular localization of LINC02892 transcripts. (B) qRT-PCR assay following nuclear, cytoplasmic and exosome fractionation detecting the distribution of the indicated LINC02892 transcripts in HT29 and SW48 cell lines. The qRT-PCR data, represented as a percentage of the total amount of detected transcripts, are presented as means ± SD from three independent experiments performed in triplicate. (C and D) Fluorescence microscopy of HT29 cells that had been transfected with the indicated plasmid (scale bars, 100 μm)

In-silico subcellular localization revealed cytoplasmic, dual nuclear/cytoplasmic, and exosomal distributions for transcript #1, #2, and #3, respectively (Fig. 3 A).

Moreover, to determine the cellular localization of the LINC02892 transcripts, the nuclear, cytoplasm, and exosome RNAs from the HT29 and SW48 cell lines were isolated, and the expression of lncRNA- LINC02892 transcripts in all subcellular locations were measured. Glyceraldehyde-3-phosphate dehydrogenase (*GAPDH*), small nuclear RNA U1 (*U1*), and *BCAR4* lncRNA were utilized as controls for cytoplasm, nucleus, and exosome, respectively. The RT-qPCR data of cellular fractionation assay in both cell lines demonstrated that the distribution of LINC02892 transcripts were clearly similar to that of the nuclear-localized *U1* snRNA, the exosomal retained *BCAR4* mRNA, and the protein-coding *GAPDH* mRNA (Fig. 3B).

To characterize the lncRNA that is enriched in the exosomes from the cell line, the extracted exosomes were examined and confirmed by TEM (data not shown).

### Protein coding potentiality

The coding potential calculator tools predicted that LINC02892 displayed no protein-coding potentiality. A protein’s potential score of transcripts was less than zero, which meant that the transcript has no capacity for coding a protein. Furthermore, the coding potential analysis revealed that LINC02892 sequence could not code any proteins. Although UniProt showed a putative peptide prediction of 28 amino acids for LINC02892 transcript #1, the putative ORF of LINC02892 transcript #1 was not expressed as an N-terminal enhanced green fluorescent protein fusion protein (Fig. 3 C and 3D).

### LINC02892 is upregulated in colorectal cancer tissue and cell lines

RNA-Seq data analysis indicated that the LINC02892 expression level was significantly high in tumorous tissues compared with adjacent normal tissues. To further confirm this observation, we obtained 40 FFPE CRC tumors and their adjacent normal FFPE tissues from CRC patients. LINC02892 expression was examined by RT-qPCR and its upregulation was observed in tumoral samples. The RT-qPCR results demonstrated that in FFPE samples, CRC tissues indicated a significant 5.11-fold overexpression of the LINC02892 as compared to the corresponding normal tissues (p-value < 0.005) (Supplementary Fig. 2). Moreover, we profiled LINC02892 expression in CRC cell lines (Caco-2, HCT 116, HT-29, SW480, and SW48) and found that the recent lncRNA ubiquitously was overexpressed in all tested CRC cell lines with higher levels compared to the normal cell line. These findings confirmed the RNA-Seq results derived from the NCBI SRA database.

## Discussion

Over the past decade, lncRNAs have been identified as significant players in gene regulation. They are often differentially expressed and widely associated with a majority of cancer types [51]. In a wide number of biological functions such as apoptosis, lncRNAs have been involved, and their roles are strongly associated with the cellular compartments where they are located [52]. Previous studies have shown that by acting as tumor suppressors or oncogenes, lncRNAs have significant roles in cancer [53]. Emerging research has indicated that DNA methylation is a significant epigenetic regulator of lncRNA expression, and the expression pattern of lncRNAs can be affected by epigenetic changes in DNA methylation which could lead to carcinogenesis [54–58].

The most abundant RNA modification in eukaryotic cells is N6-methyladenosine (m6A) [59]. RNA methylation usually occurs at the RRm6ACH consensus motif ([G/A/U][G/A]m6AC[U/A/C]) [60, 61] and is abundant in 3’ untranslated regions (3’UTRs), between stop codons and within long internal exons [62, 63]. In addition, in precursor mRNAs (pre-RNAs) and lncRNAs, m6A modification occurs [64, 65]. Proteins that can add, remove, or recognize m6A-modified sites and change substantial biological processes are m6A “writers,” “erasers” and “readers”, respectively [61]. Moreover, DNA methylation depends upon DNA methyltransferases (DNMTs) [66].

For DNA methylated in CpG islands, there are proteins called “Methyl-CpG-binding domains (MBDs)” which are required for binding to methylated DNA [67]. MBD can also bind up with RNA and influence the methylation of DNA [68]. Hence, some RNAs could direct DNA methylation. MiRNA could also influence the methylation of mRNA [69] and thus, RNA directing RNA methylation also exists. However, DNA causing RNA methylation has not been explored yet.

In the current study, an integrated methylation and transcriptome analysis was conducted to identify the crosstalk between DNA methylation and lncRNA. We identified an intergenic lncRNA based on methylation characteristics. During the past decade, due to the development of relevant biotechnology and computational methods, a growing number of newly detected lncRNAs have been reported [70]. To discover lncRNAs, there are two common methods: (1) RNA sequencing (RNA-Seq) using next-generation sequencers and (2) microarrays [71]. Owing to the development of NGS technology, lncRNA identification is now more easily achievable and several assay-based sequencing protocols have been developed to predict lncRNAs [72]. However, the identification of lncRNA relying only on RNA-Seq or microarray has some limitations. Firstly, their data are predictive and secondly, since the expression of lncRNAs are mostly low, they could be lost during normalization and trimming of the data or become absent in RNA sequencing of numerous samples. Furthermore, more complementary techniques are needed to identify the potential lncRNAs.

Since intergenic hypomethylation is crucial in tumorigenesis, aberration methylation of single nucleotide CpG sites could act as a landmark to discover long intergenic non-protein coding RNAs. It has been reported that lncRNAs are often located at crucial sites including regions of SNPs, amplifications, or common breakpoints [73], and intergenic regions [74]. Several studies have indicated that lncRNAs SNPs can prone the patients to CRC via deregulation of downstream pathways, proposing polymorphisms as CRC risk factors [8].

The DMR of DNA in intergenic regions could be related to the expression of intergenic ncRNAs [75]. Once the methylation statuses of single nucleotide CpG sites throughout the DNA genome are determined, they could be easily validated by MS-HRM. Then, the existence of a potential ncRNA could be investigated in RNA-Seq datasets as well as in-silico studies. Unlike other ncRNAs, lncRNAs are not quite conserved between species [76], causing annotation less informative in lncRNA discovery. To further confirm, gene expression should be conducted on cancer and normal tissues.

## Conclusion

In summary, based on our discovery platform, we found a novel antisense lncRNA named “LINC02892”, which has three transcripts with no capacity of coding a protein that exhibits nuclear, cytoplasmic, or exosome distributions.

Our study characterized the crosstalk between DNA methylation and lncRNA, providing a novel pipeline to identify intergenic lncRNAs like LINC02892 which could be important in tumorigenesis of CRC. Further studies are necessary to validate the efficiency of this new method.

## Electronic supplementary material

Below is the link to the electronic supplementary material.

**Supplementary Figure 1.** Methyl Specific High Resolution Melting peaks of CpG epimutation in chromosome21 analysis, normal samples (blue) and CRC patients (red).

**Supplementary Figure 2.** Real time-PCR analysis of LINC02892 gene expression in patients with CRC and normal (control) FFPE tissues (p-value <0.005). The error bars represent standard deviation (SD).

## Data Availability

The authors declare that the datasets on which the conclusions of this manuscript rely on are deposited in publicly available repositories.
