# Supplementary figures and images for: Identification of long non-coding RNA using single nucleotide epimutation analysis: a novel gene discovery approach
Source: Cancer Cell Int. 2022 Nov 4;22:337. doi: 10.1186/s12935-022-02752-2 (PMC9636742; doi:10.1186/s12935-022-02752-2)

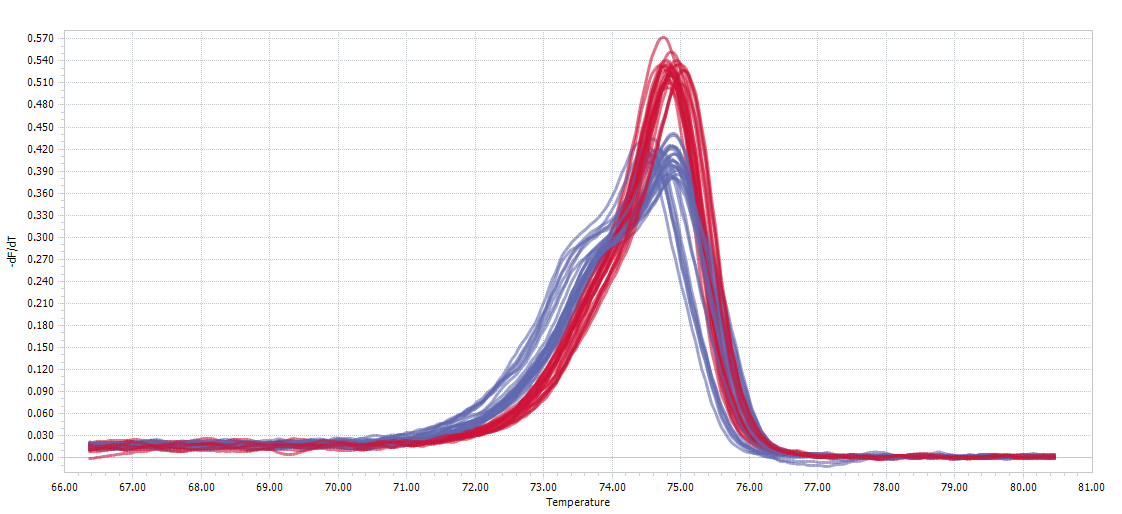

Supplement: Supplementary file 1 — Supplementary Figure 1. Methyl Specific High Resolution Melting peaks of CpG epimutation in chromosome21 analysis, normal samples (blue) and CRC patients (red). [file 12935_2022_2752_MOESM1_ESM.png]

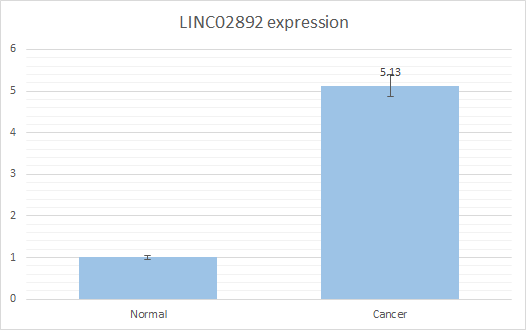

Supplement: Supplementary file 2 — Supplementary Figure 2. Real time-PCR analysis of LINC02892 gene expression in patients with CRC and normal (control) FFPE tissues (p-value <0.005). The error bars represent standard deviation (SD). [file 12935_2022_2752_MOESM2_ESM.tif]
